# Mediator Med23 deficiency in smooth muscle cells prevents neointima formation after arterial injury

**DOI:** 10.1038/s41421-021-00285-y

**Published:** 2021-08-03

**Authors:** Xiaoli Sun, Jing-wen Yin, Yan Liang, Chonghui Li, Pingjin Gao, Ying Yu, Gang Wang

**Affiliations:** 1 State Key Laboratory of Genetic Engineering, School of Life Sciences and Zhongshan Hospital, Fudan University, Shanghai, China; 2 Institutes of Biomedical Sciences, Shanghai Xuhui District Central Hospital, Zhongshan Xuhui Hospital, Fudan University, Shanghai, China; 3 Molecular and Cell Biology Laboratory, Salk Institute for Biological Studies, La Jolla, CA USA; 4 Department of Medicine, University of California-San Diego, La Jolla, CA USA; 5 State Key Laboratory of Cell Biology, Center for Excellence in Molecular Cell Science, Shanghai Institute of Biochemistry and Cell Biology, Chinese Academy of Sciences, University of Chinese Academy of Sciences, Shanghai, China; 6 International Peace Maternity and Children Hospital of China Welfare Institution, School of Medicine, Shanghai Jiao Tong University, Shanghai, China; 7 Department of Pharmacology, School of Basic Medical Sciences, Tianjin Medical University, Tianjin, China

**Keywords:** Mechanisms of disease, Transcriptional regulatory elements

Dear Editor,

Vascular smooth muscle cells (VSMCs) that line the arteries and veins, are able to increase their proliferation rate greatly following vessel wall injury and in atherogenesis. Changes in VSMC proliferation rate and differentiation state have been proposed as required procedures of injury repair. The regulation of the balance between proliferation and differentiation is of key importance in vascular biology. Our previous works have shown that Mediator MED23 subunit restricts smooth muscle cell lineage development and promotes growth related gene expression^1^. Recently, we investigated the roles of Med23 in VSMC proliferation and differentiation as well as injury-induced neointima formation by generating and examining the smooth muscle specific *Med23*-knockout (*Med23*^*sm−/*^^−^) mice. Our results showed that Med23 regulates the balance of VSMC growth and differentiation in mouse aorta. The *Med23*^sm^^−^^/^^−^ mice showed enlarged lumen and impaired contractility of aorta. RNA profiling of the aorta samples revealed that gene expression programs switching between proliferation and differentiation upon the presence or absence of Med23. More importantly, we found that *Med23* deficiency prevented neointima formation after vascular injuries, through repressing VSMC proliferation. Collectively, our data demonstrate that *Med23* has positive effects on VSMC plasticity and plays a novel pathological role in vascular injury induced neointima formation through promoting the proliferation and growth of VSMCs, thus providing a novel mechanism understanding the VSMC plasticity and related vascular diseases.

Previously we have demonstrated that Mediator MED23 plays a repressing role in RhoA signaling pathway and smooth muscle cell fate, but acts as a positive regulator for Ras-targeted genes and the adipocyte fate^1^. Nevertheless, the roles of Med23 in regulating smooth muscle development and function have not been examined in vivo in mice. Since homogenous knockout of *Med23* is embryonic lethal^2^, we generated smooth muscle specific *Med23*-knockout (*Med23*^*sm−/−*^) mice to investigate the in vivo function of Med23. We crossed conditional *Med23-*knockout mice (*Med23*^*fl/fl*^)^3^ with the aortic smooth muscle actin (*Acta2*)-Cre expressing line^4^ to generate the *Med23*^*sm−/−*^ mutant mice. Western blot analysis of tissue samples from *Med23*^*sm−/−*^ mice showed specific and efficient Med23 ablation in stomach and aorta, the walls of which are lined with smooth muscle, but not in kidney, spleen, liver, fat tissue or lung (Fig. 1a). Note that Med23 protein level in aorta from control mice was low, suggesting a repressing function of Med23 during smooth muscle cell (SMC) differentiation, whereas essentially no Med23 protein was detected in the aorta from *Med23*^*sm−/−*^ mice (Fig. 1a). Western blot analysis of isolated VSMCs from the aortas confirmed *Med23* deletion in the VSMCs from the aortas of *Med23*^*sm−/−*^ mice (Supplementary Fig. S1f).

**Fig. 1:**
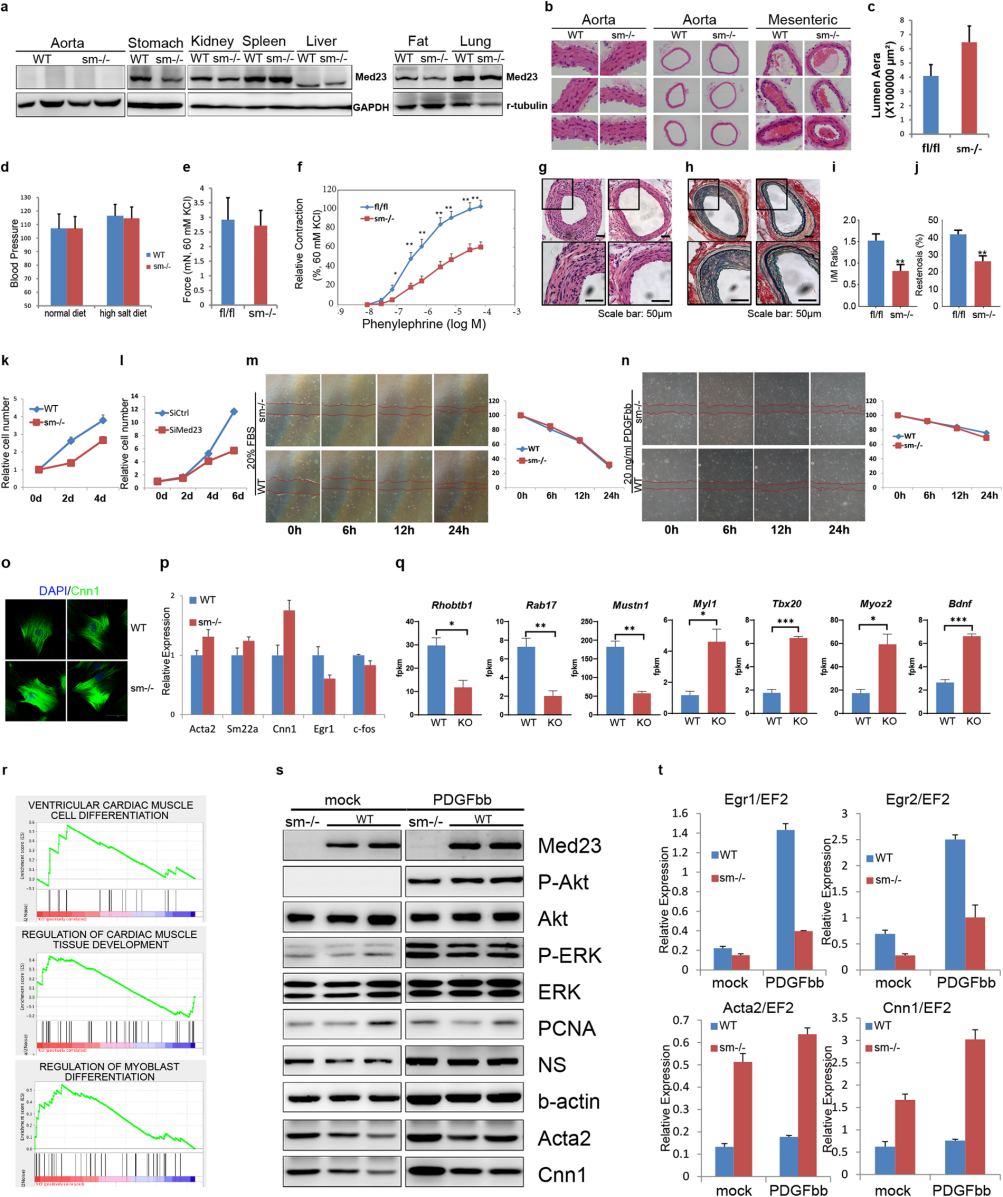
Med23 deficiency in smooth muscle cells prevents neointima formation after arterial injury. **a** Immunoblot of Med23 in various tissues isolated from *Med23*^sm−/−^ and control mice. **b**, **c** Aorta, mesenteric arteries (**b**) and aortic lumen area (**c**) in *Med23*^sm−/^^−^ and control mice. **d** Blood pressure of *Med23*^*sm−/*^^−^ and control mice on normal or high salt diets. **e** Isometric contraction of aorta rings prepared from *Med23*^*sm−/*^^−^ and control mice (60 mM KCl). **f** Dose-response curves for PE-induced contraction in *Med23*^*sm−/*^^−^ and control aortas (60 mM KCl). **g** Representative H/E staining of femoral arteries from *Med23*^*sm−/−*^ and control mice. **h** Representative elastin staining of femoral arteries from *Med23*^*sm−/*^^−^ and control mice. **i**, **j** Intima-to-media ratio (**i**) and restenosis index (**j**) of femoral arteries harvested from *Med23*^*sm−/−*^ and control mice. **k** Cell proliferation of VSMCs isolated from the aorta. **l** Cell proliferation of A7R5 cells after viral-mediated siRNA knockdown of *Med23*. **m**, **n** Wound healing assay in the presence of either 20% FBS (**m**) or 20 ng/ml PDGFbb (**n**). **o** Immunostaining of *Med23*^*sm−/*^^−^ VSMCs. **p** Expression levels of SMC genes and growth-related genes in the isolated VSMCs. **q**, **r** RNA-seq analysis of the aorta samples from *Med23*^*sm−/*^^−^ mice. **s**, **t** Western blot (**s**) and real-time PCR (**t**) of isolated VSMCs from control and *Med23*^*sm−/*^^−^ mice. NS, nucleostemin. Data are presented as means ± SEM. *P < 0.05, **P < 0.01, ***P < 0.001, vs control.

*Med23*^*sm−/*^^−^ mice were born at the expected Mendelian ratio, and they appeared to be normal and indistinguishable from their wild-type littermates. Careful analysis of the aortas revealed the differences between control and *Med23*^*sm−/−*^ mice: *Med23*^*sm−/−*^ mice exhibited slightly dilated aortas (Fig. 1b, left and middle panels) and enlarged aorta lumen (Fig. 1c) compared with control mice. In spite of this, blood pressure of *Med23*^*sm−/−*^ mice was comparable to control mice on either normal or high salt diets (Fig. 1d). We then examined the mesenteric arteries, since the mesenteric arterial network that carries about one fourth of the blood pumped from heart contributes significantly to vascular resistance and blood pressure^5^. Consistently, the morphology of mesenteric arteries was similar between *Med23*^*sm−/−*^ mice and control mice (Fig. 1b, right panels).

Next, we measured the isometric contraction of aortas. Compared to control mice, the contractility of aorta rings from *Med23*^*sm−/−*^ mice was only slightly decreased in high-K^+^ medium (Fig. 1e). However, dose-response curves for the effect of phenylephrine (PE), an α-adrenergic receptor agonist that stimulates blood vessel contraction by activating postsynaptic adrenergic receptors, showed significant reduced levels of PE-induced contraction in *Med23*-depleted aortas (Fig. 1f). Collectively, *Med23*^*sm−/−*^ mice show impaired aortic vasoconstriction, especially in response to PE.

The enlarged lumen and impaired contractility of aortas from *Med23*^*sm−/−*^ mice led us to investigate whether these mice possess other changes in vascular function. Neointima formation, which causes unfavorable restenosis after endovascular interventions such as stenting or angioplasty, is a pathological healing response induced by vessel wall injuries. We examined the occurrence of neointima formation in *Med23*^*sm−/−*^ mice in a femoral artery wire injury model^6^. Four weeks after the injury, the femoral arteries from control mice showed typical neointima formation, in which smooth muscle cells and their extracellular matrix deposits came out inside the internal elastic lamina and evade the lumen area of the blood vessel (Fig. 1g, h, left panels). Interestingly, we observed greatly reduced level of neointima formation in femoral arteries harvested from *Med23*^*sm−/−*^ mice (Fig. 1g, h, right panels). Consistently, the femoral arteries harvested from *Med23*^*sm−/−*^ mice showed substantial decrease of both intima-to-media (I/M) ratio (defined as the intimal area divided by the medial area that was calculated as the area encircled by the external elastic lamina minus the intima area, Fig. 1i) and restenosis index (percentage of luminal narrowing, defined as the intimal area divided by the area encircled by the internal elastic lamina, Fig. 1j), in comparison to those from WT mice, which demonstrated ample neointima formation. Therefore, *Med23* deficiency represses neointima formation that contributes to restenosis.

Studies indicate that medial SMCs will be activated and start to replicate and migrate in response to vessel wall injury^7,8^. With the stimulating from local growth factors, such as platelet-derived growth factor (PDGF)^9^, SMCs migrate from tonica media to intima, and proliferate further there. Although the proliferation of VSMC plays a central role in injury repair, excess proliferation of VSMC may lead to arterial injury induced neointima formation. In order to investigate the cellular mechanism behind the reduced neointima formation in *Med23*^*sm−/−*^ mice after arterial injury, we examined cell proliferation and migration of VSMCs isolated from the aorta (Fig. 1k–n). Comparing to WT VSMCs, VSMCs from *Med23*^*sm−/−*^ mice showed reduced rate of cell proliferation during the 4 days period assayed (Fig. 1k). Consistently, viral-mediated siRNA knockdown of *Med23* in a rat smooth cell line, A7R5, led to reduced cell proliferation rate (Fig. 1l) and lower DNA replicating rate, indicated by EdU incorporation (Supplementary Fig. S1a–c). Whereas in the wound healing assays stimulated with either 20% FBS or 20 ng/ml PDGFbb, VSMCs from *Med23*^*sm−/−*^ mice were able to cover the wounded area to the same extend as those from control mice (Fig. 1m, n). siMed23 A7R5 cells also showed similar migration ability as control cells in the wound healing assay (Supplementary Fig. S1d, e). Since cell proliferation, migration and increased cell size all contributed to wound healing, we next performed transwell assay to specifically measure the migration capacity of *Med23*^*sm−/−*^ VSMCs. Note that both wound healing assay and transwell assay measure cell migration in vitro, which may not reflect cell migration capacity in vivo. However, transwell assay is closer to migration models. We found that VSMCs from *Med23*^*sm−/−*^ mice were able to migrate as fast as those from control mice (Supplementary Fig. S1f, g). These results indicate that the reduced neointima formation observed in *Med23*^*sm−/−*^ mice is not caused by the reduced ability of VSMCs to migrate to intima, but rather caused by the reduced proliferation rate of VSMCs in the absence of *Med23*.

Comparing with many other terminal differentiated cell types, one special character of VSMC is its plasticity of switching between differentiation and growth state, which is essential for vessel wall injury repairing, restenosis, atherosclerosis, etc. The expression levels of *Med23* in normal aorta samples are already very low (Fig. 1a), suggesting that *Med23* level may be repressed in VSMCs to maintain the differentiated contractile phenotype. To look through the growth and differentiation of VSMCs, we examined the morphology and gene expression of isolated VSMCs from *Med23*^*sm−/−*^ mice. First, western blot analysis confirmed specific and efficient *Med23* deletion in VSMCs isolated from the aorta of *Med23*^*sm−/−*^ mice (Supplementary Fig. S1f). SMC marker CNN1 is a microfilament-associated protein that functions in the contraction of smooth muscles^10^. Immunostaining revealed increased level of Cnn1, which forms highly organized stress fibers that may alter cell contraction properties, in *Med23*^*sm−/−*^ VSMCs comparing to control cells (Fig. 1o). In addition to Cnn1, the expression levels of SMC genes such as *Acta2* and *Sm22a* were increased in the isolated *Med23*^*sm−/−*^ VSMCs, whereas the expression levels of growth-related genes like *Egr1* and *c-fos* were decreased (Fig. 1p), which is consistent with the genes regulated by *MED23* in a mesenchymal stem cell line and adipose-derived stem cells^1^. To further investigate the changes in gene expression profiling associated with *Med23* ablation, we performed RNA-seq analysis of the aorta samples from *Med23*^*sm−/−*^ mice (Fig. 1q, r). We found that *Rab17* (a member of *RAS* oncogene family), *Rhobtb1* (Rho-related BTB domain containing 1) and musculoskeletal gene *Mustn1* were among the down-regulated genes in *Med23*^*sm−/−*^ samples, in addition to *Egr1*. While the expression of muscle-related genes *Myl1*, *Tbx20*, *Myoz2* and brain derived neurotrophic factor (*Bdnf)* were up-regulated in *Med23*^*sm−/−*^ samples (Fig. 1q). Taken together, our data indicate that *Med23* deletion affects the balance between growth and differentiation of VSMCs in mouse aorta, with growth related genes down-regulated and SMC lineage-related genes up-regulated.

PDGF induces SMC migration and further proliferation, and represses smooth muscle differentiation, by specifically suppressing the expression of contractile protein genes, inducing the phosphorylation of Elk-1 and increasing the association of Elk-1 with SRF^11^. In order to examine whether Med23 promotes cell proliferation via upregulating PDGF signaling, we compared the downstream effectors of PDGFbb in *Med23*^*sm−/−*^ VSMCs and control cells. Upon PDGFbb stimulation, VSMC samples from both control and *Med23*^*sm−/−*^ mice showed significantly increased levels of phosphorylated ERK (p-ERK) and phosphorylated Akt (p-Akt) (Fig. 1s). Therefore, *Med23* deletion does not affect the downstream signaling of PDGFbb. In the absence of PDGFbb, the expression levels of growth-related genes *Egr1* and *Egr2* were slightly lower in *Med23*^*sm−/−*^ samples than in WT samples. *Egr1* and *Egr2* exhibited huge increases in expression levels in WT samples upon PDGFbb stimulation, in contrast to only moderate increase in *Med23*^*sm−/−*^ samples (Fig. 1t). On the other hand, both the RNA and protein levels of muscle lineage genes *Acta2* and *Cnn1* were significantly higher in *Med23*^*sm−/−*^ samples than in WT samples in the absence of PDGFbb (Fig. 1s, t). Upon PDGF stimulating, the expression of *Acta2* and *Cnn1* further increased in *Med23*^*sm−/−*^ samples but remained at low levels in control samples (Fig. 1t). These results indicate that the reduced neointima formation in *Med23*^*sm−/−*^ mice is not caused by defects in injury-induced signal transductions of SMC, but rather caused by the changes in transcription of SMC growth and differentiation genes, which is specifically controlled by Med23 subunit of the Mediator.

Taken together, our results suggest that *Med23* is important for the function of smooth muscle in vivo, through maintaining the balance of SMC growth and differentiation. Moreover, *Med23* is involved in neointima formation induced by arterial injury, which may improve our understanding on vascular diseases.

## Supplementary information

Supplementary Information
