# Author Correction: Mediator Med23 deficiency in smooth muscle cells prevents neointima formation after arterial injury

**DOI:** 10.1038/s41421-023-00594-4

**Published:** 2023-08-28

**Authors:** Xiaoli Sun, Jing-wen Yin, Yan Liang, Chonghui Li, Pingjin Gao, Ying Yu, Gang Wang

**Affiliations:** 1 State Key Laboratory of Genetic Engineering, School of Life Sciences and Zhongshan Hospital, Fudan University, Shanghai, China; 2 Institutes of Biomedical Sciences, Shanghai Xuhui District Central Hospital, Zhongshan Xuhui Hospital, Fudan University, Shanghai, China; 3 Molecular and Cell Biology Laboratory, Salk Institute for Biological Studies, La Jolla, CA USA; 4 Department of Medicine, University of California-San Diego, La Jolla, CA USA; 5 State Key Laboratory of Cell Biology, Center for Excellence in Molecular Cell Science, Shanghai Institute of Biochemistry and Cell Biology, Chinese Academy of Sciences, University of Chinese Academy of Sciences, Shanghai, China; 6 International Peace Maternity and Children Hospital of China Welfare Institution, School of Medicine, Shanghai Jiao Tong University, Shanghai, China; 7 Department of Pharmacology, School of Basic Medical Sciences, Tianjin Medical University, Tianjin, China

**Keywords:** Mechanisms of disease, Transcriptional regulatory elements

Correction to: *Cell Discovery* (2021) 7:59

10.1038/s41421-021-00285-y published online 03 August 2021

The authors apologize for an error in Fig. 1s. The image of Cnn1 is a duplication of the image of Acta2. The corrected Fig. 1 is shown below.

**Fig. 1:**
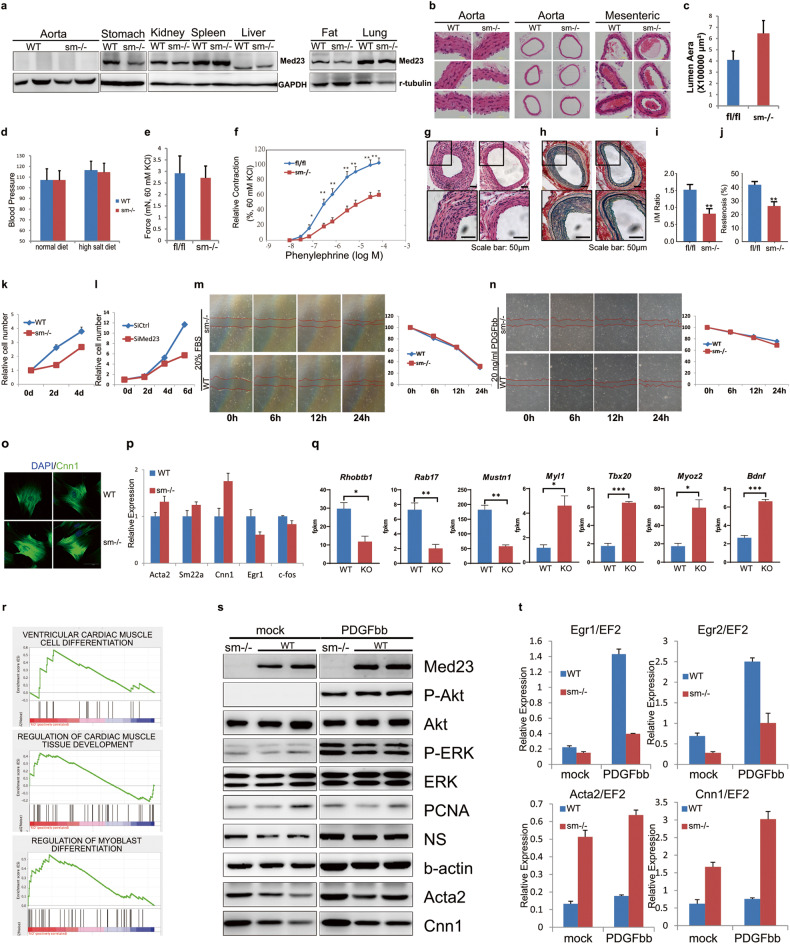
Med23 deficiency in smooth muscle cells prevents neointima formation after arterial injury. **a** Immunoblot of Med23 in various tissues isolated from *Med23*^sm−/−^ and control mice. Aorta, mesenteric arteries (**b**) and aortic lumen area (**c**) in *Med23*^sm−/−^ and control mice. **d** Blood pressure of *Med23*^sm−/−^ and control mice on normal or high salt diets. **e** Isometric contraction of aorta rings prepared from *Med23*^sm−/−^ and control mice (60 mM KCl). **f** Dose-response curves for PE-induced contraction in *Med23*^sm−/−^ and control aortas (60 mM KCl). **g** Representative H/E staining of femoral arteries from *Med23*^sm−/−^ and control mice. **h** Representative elastin staining of femoral arteries from *Med23*^sm−/−^ and control mice. Intima-to-media ratio (**i**) and restenosis index (**j**) of femoral arteries harvested from *Med23*^sm−/−^ and control mice. **k** Cell proliferation of VSMCs isolated from the aorta. **l** Cell proliferation of A7R5 cells after viral-mediated siRNA knockdown of *Med23*. Wound healing assay in the presence of either 20% FBS (**m**) or 20 ng/mL PDGFbb (**n**). **o** Immunostaining of *Med23*^sm−/−^ VSMCs. **p** Expression levels of SMC genes and growth-related genes in the isolated VSMCs. **q**, **r** RNA-seq analysis of the aorta samples from *Med23*^sm−/−^ mice. Western blot (**s**) and real-time PCR (**t**) of isolated VSMCs from control and *Med23*^sm−/−^ mice. NS, nucleostemin. Data are presented as means ± SEM. *P < 0.05, **P < 0.01, ***P < 0.001, vs control.

