# Supplementary material for: Mediator Med23 deficiency in smooth muscle cells prevents neointima formation after arterial injury
Source: Cell Discov. 2021 Aug 3;7:59. doi: 10.1038/s41421-021-00285-y (PMC8329203; doi:10.1038/s41421-021-00285-y)
Supplement: Supplementary file 1 — Supplementary Information [file 41421_2021_285_MOESM1_ESM.pdf]

Mediator Med23 Deficiency in Smooth Muscle Cells Prevents Neointima Formation after Arterial Injury

Xiaoli Sun[1,2#], Jing-wen Yin[3#], Yan Liang[4], Chonghui Li[5], Pingjin Gao[6], Ying Yu[7], Gang Wang[1*]

[1]State Key Laboratory of Genetic Engineering, School of Life Sciences and Zhongshan Hospital, Fudan University, Shanghai, China

[2]Institutes of Biomedical Sciences, Shanghai Xuhui District Central Hospital, Zhongshan Xuhui Hospital, Fudan University, Shanghai, China

[3]Molecular and Cell Biology Laboratory, Salk Institute for Biological Studies, 10010 North Torrey Pines Road, La Jolla, CA 92037, USA

[4]Department of Medicine, University of California-San Diego, 9500 Gilman Drive, La Jolla, CA 92093-0613, USA

[5]State Key Laboratory of Cell Biology, Center for Excellence in Molecular Cell Science, Shanghai Institute of Biochemistry and Cell Biology, Chinese Academy of Sciences, University of Chinese Academy of Sciences, Shanghai 200031, China

[6]International Peace Maternity and Children Hospital of China Welfare Institution, School of Medicine, Shanghai Jiao Tong University, Shanghai, China

[7]Department of Pharmacology, School of Basic Medical Sciences, Tianjin Medical University, Tianjin, China

# These authors contributed equally: Xiaoli Sun, Jing-wen Yin.

* Correspondence: Gang Wang (gwang_fd@fudan.edu.cn); ORCID:0000-0002-4582-501X

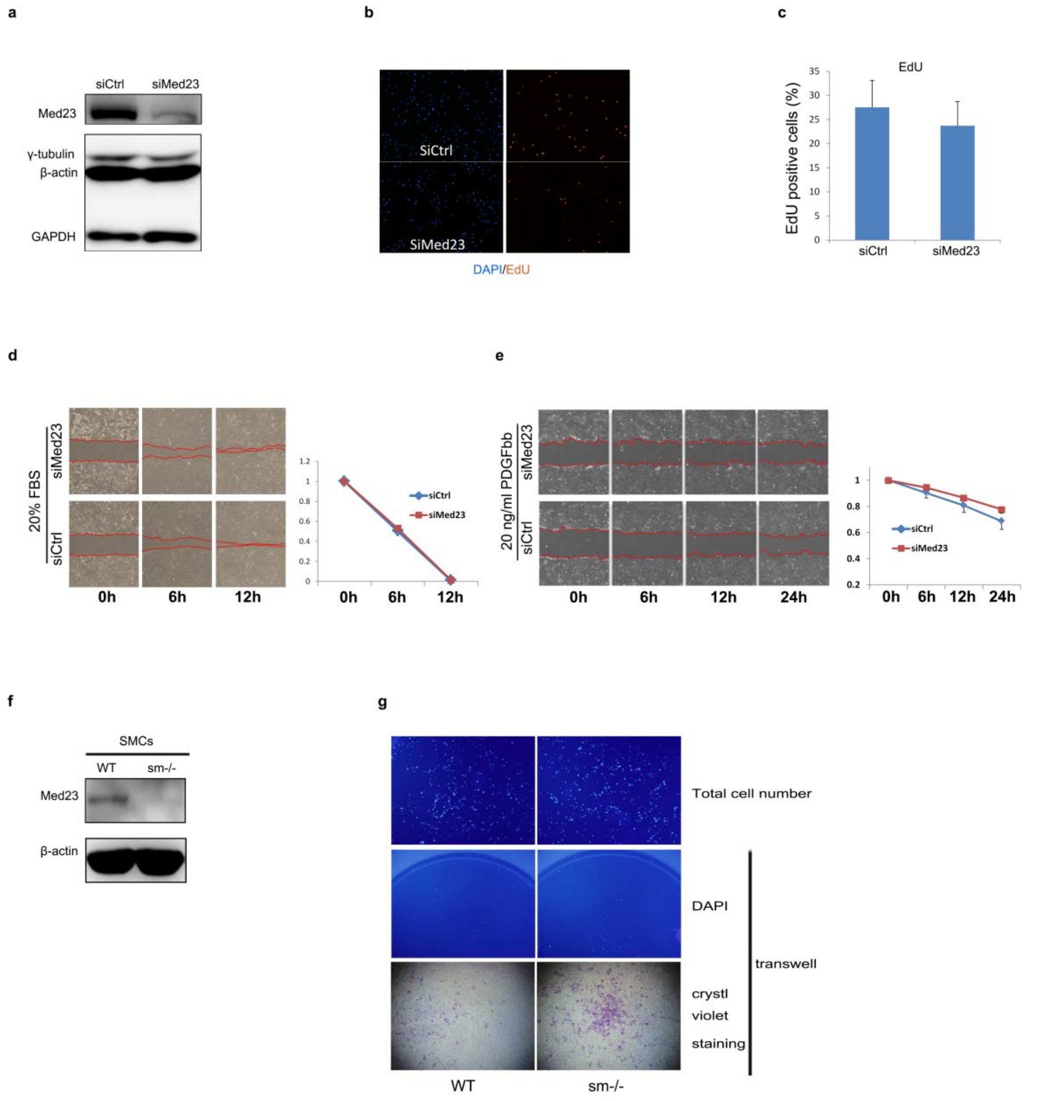

Supplementary Fig. S1 Med23 deficiency in smooth muscle cells prevents neointima formation after arterial injury. a Immunoblot of Med23 in A7R5 cells after viral-mediated siRNA knockdown of Med23. **b-c** EdU assay in siMed23 and control A7R5 cells. **d-e** Wound healing assay of siMed23 and control A7R5 cells in the presence of either 20% FBS (d) or 20 ng/ml PDGFbb **(**e**). f** Immunoblot of Med23 in vascular SMCs isolated from the aorta of *Med23*$^{sm-/-}$ mice. **g** Transwell assay in vascular SMCs isolated from the aorta of *Med23*$^{sm-/-}$ mice. Data are presented as mean ±SEM.

Materials and Methods

Animals and treatment

Med23 Floxed mice and smooth muscle aortic alpha-actin (Acta2)-Cre transgenic mice have been described previously[1,2]. Briefly, exons 5-7 were flanked by two loxP sites and this plasmid was delivered to the embryonic stem cells from Sv129 strain for homologous recombination mediated gene targeting. Smooth muscle specific Med23 knockout mice (SMKO, $Med23^{f/f}$; $Acta2\text{-}Cre^{+}$) was obtained by crossing female $Med23^{f/+}$ mice with male $Acta2\text{-}Cre^{+}$ mice. Littermates of the genotype $Med23^{f/f}$; $Acta2\text{-}Cre^{-}$ or $Med23^{+/+}$; $Acta2\text{-}Cre^{+}$ were used as controls. All mice were maintained in a specific pathogen-free facility in the Shanghai Laboratory Animal Center (Chinese Academy of Sciences) and were genotyped by PCR analysis of genomic DNA. For chow diet, 8-12-week old mice were used for experiments. For high salt diet (8% NaCl; Research Diet), mice were fed from the age of 4 weeks to 3 months. All animal experiments were conducted in compliance with National Institutes of Health guidelines and were approved by the institutional animal care and use committee of the Shanghai Institutes for Biological Sciences (Chinese Academy of Sciences). The primers for the genotyping are listed as below:

Med23-Forward: 5′-GCGGCCGCTATATGCACTGTTAGTGATT-3′,

Med23-Reverse: 5′-GTCGACCTTAGAAGAAAGCTCAAACAT-3′;

Acta2-Cre-Forward: 5’-GGTGTTAGTTGAGAACTGTGGAG-3’

Acta2-Cre-Reverse: 5’- ACATGTCCATCAGGTTCTTGC-3’

Protein isolation and Immunoblot analyses

Total protein extracts were prepared by homogenizing tissue (IKA) or lysing in RIPA solution (50 mM Tris, 10 mM EDTA, 150 mM NaCl, 0.25% Deoxycholic acid, 0.1% SDS, 2% NP-40 substitute, 0.01% Sodium azide). Protein lysates were separated on SDS-PAGE gels and transferred for 1 hour at 4°C on to a nitrocellulose membrane (BioRad). After blocking for an hour in 5% dry milk, membranes were incubated overnight at 4°C with the primary antibody in blocking buffer. Blots were washed and incubated with horseradish peroxidase (HRP)-conjugated secondary antibody (Jackson lab) for 1 hour at room temperature. GE ImageQuant LAS 4000 was used to detect protein signals after the incubation with an enhanced chemiluminescence reagent (Thermo Fisher Scientific). The antibodies were listed as below: Med23 antibody was from BD Biosciences. GAPDH,□β-actin, γ-tublin antibodies were from Santa Cruz Biotechnology. CNN1 and Acta2 antibodies were from Lifespan. Akt, p-Akt, Erk, p-Erk and PCNA antibodies were purchased from Cell Signaling Technology.

Histological analysis

Hearts and vessels were dissected from the mouse and were fixed overnight in 4% PFA/PBS. The fixed samples were embedded in OCT tissue tek (Sakura) or were dehydrated and then embedded in paraffin. Sections were cut between 5 and 10 μm thickness by using Leica microtome. Sections for histology were stained with kits for hematoxylin and eosin (Sigma-Aldrich) staining following the manufacturer's instructions.

Immunofluorescence analysis

Cells were fixed overnight in 4% PFA/PBS and blocked in 10% Goat serum/PBS before incubation with antibodies. The antibodies were diluted in a 2% bovine serum albumin solution in PBS. CNN1 antibody was purchased from Lifespan. Immunofluorescence images were acquired using Leica SP8 confocal microscope.

RNA isolation and semi-quantitative real time PCR

Total RNA was prepared using the Trizol Reagent (Sigma-Aldrich). The first strand cDNA was generated using PrimeScript RT Reagent Kit (Takara) according to the manufacturer's instructions. Real-time PCR was performed in triplicate using a SYBR Green PCR master mix in an Eppendorf Mastercycler. All values were normalized to the level of EF2 mRNA, which is constitutively expressed and not changed during the experiments. The primer sequences are listed as below:

**Primer sequences used for real-time PCR analysis**:	
Acta2-F	5'-ACTGGGACGACATGGAAAAG -3'
Acta2-R	5'-GTTCAGTGGTGCCTCTGTCA-3'
Sm22a-F	5'-ACCAAAAACGATGGAAACTACCG-3'
Sm22a-R	5'-CATTTGAAGGCCAATGACGTG-3'
Myl9-F	5'-ATCTGGAGGGCATGATGAAC-3'
Myl9-R	5'-CATCTCGTCCACCTCCTCAT-3'
Cnn1-F	5'-GTTGCGCTTGTCTGTGTCAT-3'
Cnn1-R	5'-GAGGCCATCCATGAAGTTGT-3'
Egr1-F	5'-CCAACATCAGTTCTCCAGCTC-3'

Egr1-R	5'-TTGCTCAGCAGCATCATCTC-3'
c-fos-F	5'-TTCCTACTACCATTCCCCAGCC-3'
c-fos-R	5'-GATCTGCGCAAAAGTCCTGTG-3'
EF2-F	5'-AAAAGTATGAGTGGGACGTTGC-3'
EF2-R	5'-CCTTGATCTCATTCAGGTACTGC-3'

Echocardiography

Adult mice were anesthetized with 5% isoflurane for 15 seconds and maintained at 0.5% isoflurane during the procedure. Echocardiography was performed by using a VisualSonics, SonoSite FUJIFILM, Vevo 2100 ultrasound system with a linear transducer 32-55MHz. Percentage fractional shortening (%FS) was used as an indicator of systolic cardiac function. Measurements of left ventricular (LV) internal diameter at end-diastole (LVIDd) and LV internal diameter at end-systole (LVIDs) were determined from the M-mode tracing.

Primary mouse vascular smooth muscle cells (VSMCs) Cultures

VSMCs were isolated from 6- to 8-wk-old mice as described[3]. In brief, aortas were excised from the mouse. The fat, adhering connective tissues and endothelial cells were removed under a dissecting microscope. The vessels were cut into small pieces (1 mm^2) and then covered by a glass cover slip. The cells were cultured in DMEM/F-12 media (Thermo Fisher Scientific) with 20% FBS at 37°C in a humidified atmosphere containing 5% CO2. Cultured aortic smooth muscle cells were routinely used between passages 2 and 3. For PDGFbb stimulation, 20 ng/ml human

recombinant PDGFbb (R&D Systems) was added to culture media for the indicated times.

A7R5 Rat Aortic Smooth Muscle Cell Cultures and Retrovirus Infection

A7R5 cells were maintained in Dulbecco's Modified Eagle's Medium (DMEM media supplemented with 10% fetal bovine serum (FBS), 150 units/ml penicillin and 150 μg/ml streptomycin (P/S). Stable cell lines with *Med23* knocked down were established following the manufacturer's recommendations (Clontech) and have been described previously[1]. Briefly, retroviruses were generated following the cotransfection of recombinant pSiren-RetroQ plasmids with pCL10A1 helper plasmid into 293T cells. Virus-containing supernatants were added to the A7R5 cells for spin infection by centrifugation at 1,258 × g at 30 °C for 1.5 h. Twenty-four hours after spin infection, A7R5 cells were selected with 50 μg/mL puromycin (Sigma-Aldrich).

Cell proliferation assay

Cells were seeded at 2×10^4 cells per well in 12-well plates. Cells were allowed to proliferate for an additional 4-6 days. Cell number was measured every other day after plating by a CYTORECON cell counter (GE Healthcare). Experiments were done in triplicate.

EdU incorporation assays were performed using EdU Cell Proliferation Detection Kits (Sigma Aldrich). Briefly, the Cells were seeded at 2×10^4 cells/well and quiesced for 48 h. Then, the cells were incubated in EdU labeling reagent and immunofluorescence assay was used for the detection of EdU incorporated into cellular DNA following the manufacturer's instructions. Total cellular nuclei were

stained with DAPI. The results were reported as a percentage of EdU-labelled cells to the total amount of cells.

Cell migration assays

For the scratch wound healing assays in VSMCs and A7R5 cells, the confluent monolayer of cells was scratched with a P200 pipette tip. Cellular progress was photographed at indicated time points after scratching and quantified. 6 fields at fixed location were selected per well to calculate cell migration. The data were collected from 3 independent experiments.

Transwell migration assays of VSMCs were performed using BD Falcon Cell culture inserts for 24-well plates with 8.0-μm pore filters. Cells (5×10^4 VSMCs) were seeded into the upper chamber of the insert for each well in DMEM/F-12 without other supplements, and the lower chamber of the plate was filled with DMEM/F-12 supplemented with 20% FBS or with 20% FBS + 20ng/ml PDGFbb. After 3 hours, the filter was fixed with cold 4% paraformaldehyde for 5 minutes and stained with 0.1% crystal violet (Sigma-Aldrich). Cells in the upper chamber were removed by scrubbing with a cotton swab, and 5-7 randomly selected fields of migrated cells through the filter were photographed and quantified. Each experiment was duplicated. Data were collected from 3 independent experiments.

Femoral artery wire injury model

Femoral arteries in mice were subjected to transluminal wire injury as described[4]. Briefly, 12 weeks male mice were anesthetized with isoflurane during surgery. The right femoral arteries were exposed using blunt dissection. A guide wire (0.38 mm in

diameter; Cook, Bloomington, IN, USA) was inserted into the arterial lumen through an arteriotomy and left in place for an additional 3 min to denude the artery. Sham surgeries were performed on the left femoral arteries of the same mouse as the control. Mice were allowed to recover and femoral arteries were harvested 4 weeks after the injury. Sections were stained with hematoxylin and eosin (H&E). The intimal area was defined as the area encircled by the internal elastic lamina minus the lumen area. The medial area was calculated as the area encircled by the external elastic lamina minus the intima area. The intima/media (I/M) ratio was calculated as the intimal area divided by the medial area. Restenosis (%) were defined as the intimal area divided by the area encircled by the internal elastic lamina.

Measurement of isometric force in aortic rings

Measurement of isometric force in mouse aortic rings was performed as described[5]. Briefly, mouse aortas were isolated and transferred to oxygenated Kreb's buffer (containing 4.7 mmol/L KCl, 118 mmol/L NaCl, 2.5 mmol/L CaCl2, 1.2 mmol/L KH2PO4, 1.2 mmol/L MgSO4, 11 mmol/L glucose, and 25 mmol/L NaHCO3). The aortas were cut into 2-mm rings and fixed on isometric force transducers (model 610 M; Danish Myo Technology) under an initial resting tension of 3 mN. After 60 min of incubation in Krebs solution at 37°C, rings contractility was tested three times in high-K+ mediums (60 mM KCl) to stabilize the contraction. A dose-response curve of phenylephrine (PHE, 0.01–100 μM) was performed to assess the vasoconstriction response. Data were recorded using the PowerLab/8sp data acquisition system (AD Instruments).

Blood Pressure Measurement

Blood pressures were measured by the tail-cuff method (BP-2000, Visitech Systems, Napa Place Apex, North California) as described previously[6].

Statistical Analysis

Statistical analyses were performed by two-tailed student t test and ANOVA for multi-comparisons. A p value <0.05 will be considered significant.

References

1. Yin, J.W., et al. Mediator MED23 plays opposing roles in directing smooth muscle cell and adipocyte differentiation. *Genes Dev.* **26**, 2192-2205 (2012).
2. LeBleu, V.S., et al. Origin and function of myofibroblasts in kidney fibrosis. *Nat Med.* **19**,1047-1053 (2013).
3. Liu, G., et al. Resolvin E1 attenuates injury-induced vascular neointimal formation by inhibition of inflammatory responses and vascular smooth muscle cell migration. *FASEB J.* **32**, 5413-5425 (2018).
4. Zhang, J., et al. Cyclooxygenase-2-derived prostaglandin E(2) promotes injury-induced vascular neointimal hyperplasia through the E-prostanoid 3 receptor. *Circ Res*. **113**, 104-114 (2013).
5. Kong, L.R., et al. Decrease of Perivascular Adipose Tissue Browning Is Associated With Vascular Dysfunction in Spontaneous Hypertensive Rats During Aging. *Front Physiol*. **9**, 400 (2018).
6. Krege, J.H., et al. A noninvasive computerized tail-cuff system for measuring blood pressure in mice. *Hypertension* **25**, 1111-1115 (1995).
